# Perisynaptic Astrocytic Processes as Communication Hubs and Early Sites of Dysfunction

**DOI:** 10.1177/10738584261445356

**Published:** 2026-05-25

**Authors:** Francesca Puletti, Isabella Tugulu, Soyon Hong

**Affiliations:** 1 UK Dementia Research Institute, Institute of Neurology, University College London, London, UK

**Keywords:** perisynaptic astrocytic processes, glia, PAPs, synapse loss, clasmatodendrosis, pathology, neuro–glia interactions, metabolism, astrocyte–microglia crosstalk

## Abstract

Astrocytes play key roles in shaping the synaptic environment, yet the cellular structures through which they interact with individual synapses remain incompletely understood. Perisynaptic astrocytic processes (PAPs) are ultrathin astrocytic leaflets that variably appose synapses and form a major structural interface between astrocytes and neuronal synapses. PAPs are best viewed as a perisynaptic configuration within a broader population of fine astrocytic protrusions, with coverage, geometry, and molecular composition varying across brain regions, developmental stages, and species. In this review, we synthesize current evidence that PAPs define local microdomains around synapses in which astrocytes sense neuronal activity and regulate the synaptic milieu. We discuss how PAP organization and plasticity influence neurotransmitter clearance, ion homeostasis, and structural remodeling at synapses. We also consider how regional differences in PAP organization may contribute to selective circuit vulnerability and how early PAP dysfunction may contribute to synaptic dysfunction in neurodegenerative disease. Finally, we highlight emerging approaches needed to resolve the structure and function of PAP at synapses in vivo and to establish causal mechanisms.

## Introduction

**Figure 1.**
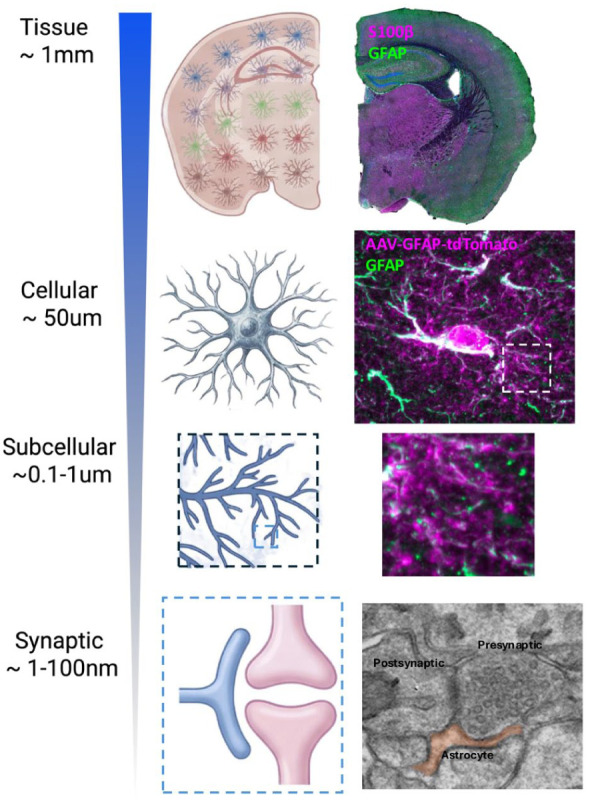
The structural limits of visualizing perisynaptic astrocytic processes (PAPs). PAPs are extremely small astrocytic structures, often <50 nm in diameter, which makes them very difficult to study with conventional imaging. Moreover, the widely used GFAP staining, as illustrated in the images, labels only primary astrocytic branches and fails to mark PAPs, leaving their structure and function largely uncharacterized in studies relying on this marker. Light microscopy images were generated by Francesca Puletti (Hong Lab). Electron microscopy images were produced in collaboration with Jemima Burden at the LMCB. (Adapted from Yu et al 2020.)

Astrocytes play central roles in shaping the synaptic environment and maintaining central nervous system (CNS) homeostasis through extensive interactions with neurons, other glial cells, and the vasculature (Liu et al 2023; Lorin et al 2024). These interactions are enabled by the highly arborized morphology of astrocytes, which allows individual cells to contact large numbers of synapses while simultaneously engaging other cellular and vascular elements of neural tissue. This structural complexity is a defining feature of astrocytes (Figure 1). In the gray matter, a major astrocyte subtype termed *protoplasmic astrocytes* extends several micrometer-scale primary branches from the soma that further divide into finer submicrometer branchlets and numerous ultrathin leaflets that permeate astrocytic territories and can be tens of nanometers in diameter (Khakh and Deneen 2019; Aten et al 2022; Salmon et al 2023; Benoit et al 2025). These terminal processes form a dense meshwork that constitutes approximately 70% to 80% of astrocyte surface area while occupying only about 10% of total cell volume (Ventura and Harris 1999; Lehre and Rusakov 2002; Semyanov and Verkhratsky 2021), enabling extensive interactions with the surrounding neuropil. A subset of these distal processes contacts the vasculature and forms specialized structures known as perivascular astrocytic processes (PvAPs), or endfeet, which are key components of the neurovascular unit and play crucial roles in neurovascular coupling and maintenance of the blood–brain barrier (Boulay et al 2017; Mazaré et al 2020).

Many of the remaining fine processes extend into the neuropil and interact with neuronal elements. A critical subset of these contacts occurs at specialized astrocytic extensions that closely appose synaptic structures, commonly referred to as perisynaptic astrocytic processes (PAPs). Synapse-associated contacts most commonly occur through ultrathin leaflets, which typically contain few organelles and represent the smallest elements of the astrocytic arbor (Ghézali et al 2016; Semyanov and Verkhratsky 2021; Aten et al 2022; Salmon et al 2023). Thicker branchlets and branches can also contact synapses, although less frequently and in a region-dependent manner. Importantly, not all leaflets are perisynaptic at any given moment. Rather, perisynaptic positioning represents a context-dependent state within a broader population of fine astrocytic protrusions (Hodebourg et al 2025). These structural distinctions are functionally relevant: the restricted volume and sparse organelle content of leaflets impose constraints on local signaling mechanisms that differ from those operating in larger astrocytic branches (Patrushev et al 2013). Fine astrocytic protrusions can also interact with other neuronal structures, including neuronal somata and nodes of Ranvier (Sakry et al 2014; Akinlaja and Nishiyama 2024), highlighting the structural diversity of astrocyte–neuronal interfaces across the neuropil.

The terminology surrounding PAPs has been used inconsistently in the literature. In earlier work, the abbreviation “PAP” was used to denote peripheral astrocyte processes more generally (Derouiche and Frotscher 2001). In this review, we use the term *perisynaptic astrocytic processes* (PAPs) to refer specifically to ultrafine astrocytic extensions within the neuropil that closely appose synaptic elements (Benoit et al 2025; Hodebourg et al 2025). At these sites, astrocytes participate in multipartite synaptic signaling hubs with variable astrocytic coverage rather than forming a uniform structural component of all synapses. In addition to astrocytes and neurons, other glial elements such as oligodendrocyte precursor cell (OPC) processes have also been reported to associate with synaptic structures (Sakry et al 2014; Akinlaja and Nishiyama 2024), further highlighting the cellular complexity of these local signaling environments. PAPs exhibit molecular and biophysical properties that differ from their parent branches (Patrushev et al 2013), and their distribution and structural relationships with synapses vary substantially across brain regions and circuits (Bernardinelli et al 2014). Such regional heterogeneity suggests that astrocyte–synapse interactions are organized according to local circuit architecture rather than following a uniform structural template.

Because astrocytes extend thousands of fine processes throughout the neuropil, many astrocyte–synapse interactions occur within highly localized compartments rather than across the cell as a whole (Benoit et al 2025). PAPs can therefore be viewed as specialized perisynaptic microdomains that support the integration of neuronal activity with metabolic and homeostatic signals. Their geometry, molecular composition, and proximity to synapses create local microenvironments where astrocytes can regulate neurotransmitter clearance, ion homeostasis, and extracellular signaling (Benoit et al 2025). These local interactions allow astrocytes to influence synaptic transmission while engaging broader cellular programs. This perspective may also help explain why astrocytes frequently exhibit immune-and stress-related gene signatures in transcriptomic studies (Habib et al 2020; Clarke et al 2023), despite not typically performing the canonical effector functions of professional immune cells. Rather than reflecting canonical immune responses, these signatures may correspond to molecular systems that enable astrocytes to detect and respond to local perturbations in synaptic or metabolic state (Lee et al 2022). Because PAPs form an interface between the synaptic cleft, the extracellular space, and the broader astrocytic arborization, they are well positioned to integrate diverse signals into spatially restricted responses (Benoit et al 2025).

At the same time, direct causal evidence for many proposed PAP functions remains limited. Our ability to manipulate these ultrafine astrocytic domains with spatial and temporal precision is still developing, and several proposed roles, including local signaling coordination or synaptic surveillance, remain incompletely tested. Current data instead suggest that PAPs exhibit structural plasticity on time scales associated with development, physiological state, or pathology, while remaining relatively stable under many baseline conditions (Bernardinelli et al 2014). In this review, we synthesize structural, molecular, and functional data describing PAPs as specialized integrative domains at the astrocyte–synapse interface. We discuss how PAP organization may shape synaptic signaling, circuit excitability, neurometabolic coupling, and microglia–synapse interactions, and we examine how alterations in these structures may contribute to circuit vulnerability in neurological disease. Finally, we highlight key open questions and emerging experimental approaches needed to resolve PAP structure and function at microdomain resolution.

## Structural and Functional Roles of PAPs in Healthy Synaptic Regulation

Understanding how these fine astrocytic processes interact with synapses is important because they regulate the local microenvironment in which synaptic transmission occurs, influencing neurotransmitter clearance, ionic balance, and structural remodeling of synaptic circuits.

### Regional Organization of Astrocyte–Synapse Contacts

Astrocytes occupy spatially defined territories that enable individual cells to interact with large numbers of synapses within local neural circuits. The scale of these interactions varies across species. In rodents, protoplasmic astrocytes contact an estimated 20,000 to 120,000 synapses, depending on brain region, whereas human astrocytes occupy substantially larger and more complex territories and may interface with up to 2,000,000 synapses (Bushong et al 2001; Oberheim et al 2009; Hercher et al 2025).

Astrocyte–synapse contacts arise from 2 structurally distinct classes of astrocytic processes. Ultrathin leaflets that contain few organelles account for most perisynaptic contacts, whereas thicker branchlets and branches containing mitochondria and other intracellular structures contact a smaller fraction of synapses (Khakh and Deneen 2019; Aboufares El Alaoui et al 2021; Semyanov and Verkhratsky 2021; Aten et al 2022; Salmon et al 2023). These morphological differences likely impose distinct constraints on local signaling mechanisms, with leaflets favoring highly localized transporter- and receptor-mediated signaling, while larger processes may support more complex intracellular signaling and metabolic functions associated with organelle-containing astrocytic compartments (Khakh and Deneen 2019; Semyanov and Verkhratsky 2021). Throughout this review, the term *PAPs* primarily refers to the fine leaflet-like astrocytic processes that contact synaptic elements, while acknowledging that thicker branchlets and branches can also interact with synapses in a region-dependent manner.

The extent of astrocyte–synapse apposition varies substantially across brain regions and circuits. Astrocytic processes rarely fully surround synapses; instead, PAPs typically form partial appositions with pre- and postsynaptic elements, with the extent of coverage varying across circuits and physiological conditions (Verkhratsky and Butt 2023). In the hippocampus, PAPs contact approximately 57% to 62% of excitatory synapses and cover 38% to 58% of their perimeter. In the cerebellum, climbing fiber synapses exhibit particularly extensive astrocytic coverage, reaching up to 87%, compared to approximately 65% for parallel fiber synapses (Ventura and Harris 1999; Bernardinelli et al 2014; Tao-Cheng 2025; Chiappini et al 2026). Neocortical coverage is more heterogeneous, typically ranging from 29% to 56%, although some cortical regions such as layer IV can approach approximately 90% coverage (Ventura and Harris 1999; Bernardinelli et al 2014). These regional differences suggest that the geometry of astrocyte–synapse interactions is shaped by local circuit architecture rather than following a uniform structural template.

Such variability in PAP coverage may influence how neurotransmitters and ions are handled within the synaptic microenvironment. Synapses with extensive astrocytic coverage are likely to experience tighter biochemical compartmentalization and more rapid neurotransmitter clearance consistent with higher local transporter density, whereas circuits with lower astrocytic coverage may permit greater extracellular diffusion of signaling molecules and support broader network-level interactions (Ghézali et al 2016).

### Developmental Emergence of Perisynaptic Astrocytic Processes

The structural organization of PAPs emerges through coordinated developmental programs that parallel synapse formation. During postnatal development, astrocytes undergo substantial morphological maturation between approximately postnatal days P7 and P28 (Bushong et al 2004; Morel et al 2014). Over this period, simple filopodia-like processes progressively elaborate into complex spongiform arbors, and astrocyte territories become largely nonoverlapping by P21–P28.

This maturation closely parallels glutamatergic synaptogenesis. Between P14 and P26, the number of VGLUT1^+^ presynaptic elements contacted by individual astrocytes increases steeply alongside expansion of astrocytic territory (Morel et al 2014; Ghézali et al 2016). Synaptically released glutamate and astrocytic mGluR5 signaling contribute to this process: genetic ablation of VGLUT1 or mGluR5 reduces astrocyte domain growth, synaptic coverage, and induction of the glutamate transporter GLT-1 (Morel et al 2014; Ghézali et al 2016; Nozawa et al 2023).

Once established, PAPs may also contribute to synapse assembly during development. The astrocyte-secreted protein hevin promotes formation of thalamocortical excitatory synapses by bridging presynaptic neurexin 1α and postsynaptic neuroligin 1 (Singh et al 2016). Additional contact-dependent mechanisms regulate astrocyte morphogenesis. Astrocytic neuroligins interact with neuronal neurexins to promote astrocyte territory growth, while δ-catenin/N-cadherin signaling contributes to layer-specific astrocyte morphogenesis in the cortex (Stogsdill et al 2017; Tan et al 2023). BDNF/TrkB.T1 signaling has also been implicated in the formation of fine astrocytic processes during critical developmental windows in motor cortex, barrel cortex, and thalamus (Pinkston et al 2024).

At the transcriptional level, extrinsic signals induce maturation-associated transcription factors such as Rorb, Dbx2, Lhx2, and Fezf2, which regulate expression of transporters and ion channels enriched in perisynaptic astrocytic domains (Lattke et al 2021). Interestingly, astrocytic endfeet associated with the neurovascular units (PvAPs) mature slightly earlier (approximately P7–P14) than neuropil-facing PAPs, suggesting that distinct developmental programs establish vascular and synaptic astrocytic compartments (Freitas-Andrade et al 2023).

### Structural Plasticity of PAPs

Although the structural organization of astrocyte–synapse contacts is shaped during development, PAPs retain the capacity for structural remodeling under specific physiological or activity-dependent conditions. Experimental manipulations such as long-term potentiation (LTP), long-term depression (LTD), sensory experience, and high-frequency stimulation can alter astrocyte–synapse apposition over time scales ranging from minutes to hours (Genoud et al 2006; Lushnikova et al 2009; Bernardinelli et al 2014; Perez-Alvarez et al 2014; Henneberger et al 2020; Nam et al 2025).

In the hippocampus, LTP is often associated with partial retraction of astrocytic processes from large dendritic spines, which may alter glutamate uptake efficiency and increase activation of extrasynaptic NMDA receptors (Nam et al 2025). Conversely, LTD has been associated with increased astrocytic coverage of synapses, potentially enhancing transmitter containment and ionic homeostasis. Structural rearrangements of astrocytic processes have also been observed in the cerebellum during excitatory stress conditions (Tao-Cheng 2025).

Behavioral and metabolic states can similarly influence astrocyte morphology. Fear learning has been associated with transient reductions in astrocytic coverage of hippocampal synapses (Mazaré et al 2020), whereas caloric restriction increases synaptic ensheathment in the hippocampus (Popov et al 2020). Sleep–wake cycles also influence astrocyte structure and metabolism. Glycogen, stored within astrocytes, is mobilized during wakefulness to generate the ATP needed for cytoskeletal remodeling and the extension of PAPs toward active synapses (Bellesi et al 2015; McCauley et al 2020). During sleep, glycogen synthesis increases, the energetic demand for process extension declines, and PAPs partially retract, thereby increasing extracellular diffusion of neurotransmitters. These dynamic changes are thought to contribute to synaptic homeostasis and memory consolidation. Together, these observations suggest that PAP organization is shaped by both circuit-level architecture and activity-dependent structural plasticity.

The closest astrocyte–synapse contacts, however, may remain relatively stable under baseline conditions. Experiments using a FRET (Förster Resonance Energy Transfer)-based approach capable of detecting astrocyte–presynaptic contacts at nanometer resolution demonstrated that these intimate interfaces remain largely unchanged during brief neuronal activation or metabolic stress in adult striatal slices, even when the overall astrocyte territory swells (Octeau et al 2018). These data suggest that astrocytic coverage may remodel with experience or pathology, while the most intimate nanoscale astrocyte–synapse contacts remain comparatively stable.

### Molecular and Cellular Mechanisms of PAP Function

**Figure 2.**
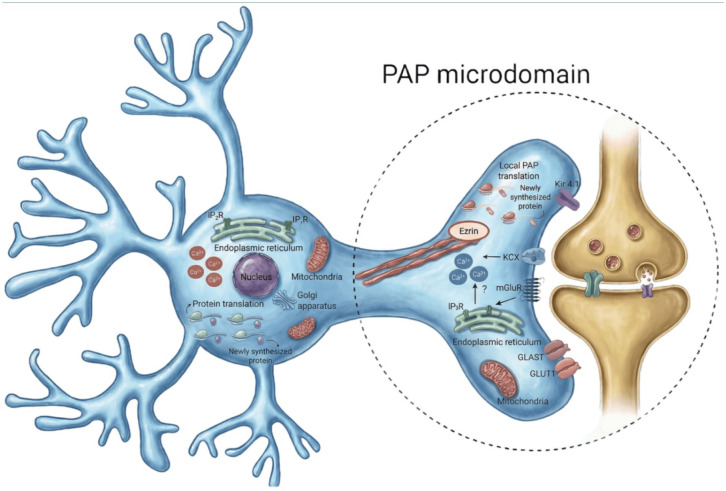
Molecular mechanisms shaping perisynaptic astrocytic processes (PAPs) dynamics and perisynaptic signaling. PAP motility is regulated by the actin–membrane linker ezrin, which is locally activated by neuronal activity and Ca^2+^ signals. Whether PAPs sustain endoplasmic reticulum (ER)–dependent IP_3_R Ca^2+^ microdomains remains debated, but they reliably generate rapid, compartmentalized Ca^2+^ events through ER-independent Na+/Ca^2+^ exchanger (NCX) reversal. Neuronal activity dynamically redistributes key membrane proteins, such as GLT-1/GLAST, via local translation and trafficking within PAPs. This activity-dependent protein synthesis (“PAPome”) rapidly adjusts cytoskeletal elements and transporter abundance. PAPs also integrate K^+^-dependent electrical responses, where presynaptic K^+^ efflux and electrogenic glutamate uptake depolarize PAPs, tuning glutamate clearance and synaptic strength.

The specialized functions of PAPs depend on molecular mechanisms that regulate their nanoscale structure, intracellular signaling, and membrane transport systems (Figure 2). At the structural level, the architecture of PAPs is supported by cytoskeletal regulators that link the plasma membrane to the underlying actin cytoskeleton. The membrane-to-actin linker ezrin, which is enriched in fine astrocytic processes, plays a key role in maintaining PAP structure (Lavialle et al 2011; Molotkov et al 2013; Bernardinelli et al 2014). Activation of ezrin through Ca^2+^-dependent phosphorylation promotes coupling between the plasma membrane and actin filaments, supporting remodeling of these ultrafine astrocytic protrusions.

The origin of Ca^2+^ signals within PAPs remains an active area of investigation. Super-resolution imaging indicates that some astrocytic leaflets contain endoplasmic reticulum (ER) elements capable of generating localized IP3-dependent Ca^2+^ transients following activation of Gq-coupled receptors such as metabotropic glutamate receptors (Lavialle et al 2011; Arizono et al 2020; Benoit et al 2025). However, computational modeling suggests that nanoscale geometry, including the degree of perisynaptic coverage, may influence the formation of Ca^2+^ microdomains, potentially limiting or shaping local signaling within tightly apposed leaflets (Toman et al 2023). Furthermore, ultrastructural studies suggest that ER structures are sparse in PAPs and present in only a subset of processes (Patrushev et al 2013; Aboufares El Alaoui et al 2021).

One explanation for this apparent discrepancy is that nanoscale organization of ER signaling domains can permit efficient Ca^2+^ release even from ER compartments, as clustering of IP_3_ receptors can locally amplify Ca^2+^ signals despite limited ER volume (Denizot et al 2022). In addition, Ca^2+^ elevations may also arise through ER-independent mechanisms. For example, Na^+^ accumulation associated with glutamate uptake can reverse the Na^+^/Ca^2+^ exchanger and generate localized Ca^2+^ elevations coupled to synaptic activity (Wade et al 2019).

Additional molecular regulators also contribute to the organization of PAPs. The astrocytic adhesion molecule Connexin-30 has been shown to regulate the structure of PAPs by limiting activity-dependent lysosomal degradation pathways, thereby influencing the stability of PAP-associated proteins (Ghézali et al 2020).

Beyond Ca^2+^ signaling, ion transport systems located in PAP membranes further support the regulatory functions of PAPs (Brazhe et al 2023; Rose and Verkhratsky 2024). Synaptic glutamate is rapidly taken up by astrocytic transporters EAAT1/2, which cotransport 3 Na^+^ ions per glutamate molecule (Herde et al 2020). This process generates a transient intracellular Na^+^ gradient that support secondary processes like neurotransmitter clearance, including those of glutamate, GABA, and glycine, as well as metabolic shuttling through glutamine recycling, glucose supply, and monoamine clearance (Roux and Supplisson 2000; Unichenko et al 2012; Rose and Verkhratsky 2024). These Na^+^ elevations can also engage the Na^+^/Ca^2+^ exchanger (NCX), providing an additional route for Ca^2+^ influx coupled to synaptic activity (Wade et al 2019).

### PAPs in Synaptic Homeostasis and Remodeling

**Figure 3.**
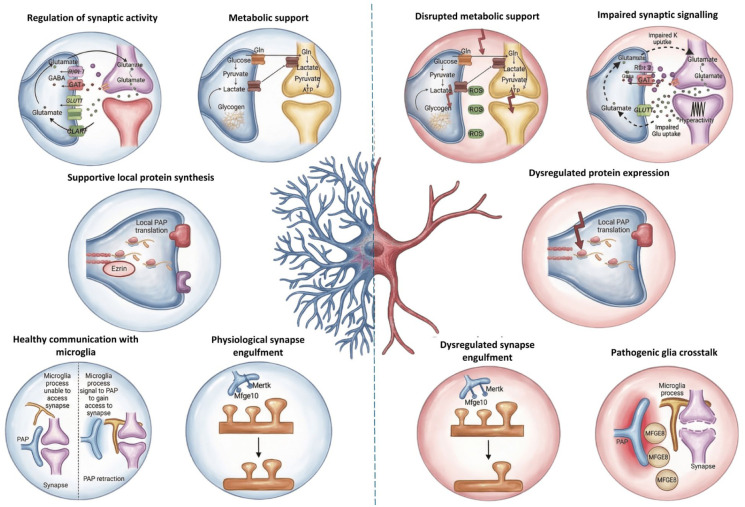
Perisynaptic astrocytic process (PAP) dynamics in healthy and pathological synaptic regulation. In the healthy brain, PAPs play a fundamental role in maintaining synaptic function by regulating ionic and neurotransmitter balance (glutamate and K^+^), supporting neuronal metabolic demands, engulfing weakened synapses, and coordinating with microglia to enable physiological synapse elimination. In pathological conditions, PAPs change their morphology, and these tightly coordinated PAP-mediated processes become dysregulated, leading to impaired synaptic homeostasis and widespread disruption of neuronal circuit function.

The molecular mechanisms described above enable PAPs to regulate the biochemical environment of synapses (Figure 3). A central function of these domains is the clearance of neurotransmitters from the synaptic microenvironment. High-affinity glutamate transporters, including GLT-1 and GLAST, are highly enriched in astrocytic membranes surrounding synapses (Rothstein et al 1994). The efficiency of glutamate uptake depends on both transporter density and the geometry of astrocyte–synapse apposition, with tighter astrocytic coverage generally associated with faster transmitter clearance (Herde et al 2020). This uptake process is supported by the highly negative membrane potential of astrocytes, maintained in part by inwardly rectifying potassium channels such as Kir4.1.

Neuronal activity can also influence synaptic signaling through electrical interactions with PAP membranes. During neuronal firing, potassium (K^+^) released into the extracellular space and the electrogenic uptake of glutamate can depolarize PAPs, reducing the driving force for glutamate transport and promoting transient glutamate spillover that enhances extrasynaptic neuronal signaling (Armbruster et al 2022; Byvaltcev et al 2023). Local neuronal mechanisms may further shape this feedback. For example, reversal of the neuronal KCC2 cotransporter has been proposed to limit presynaptic release and shorten LTP duration under these conditions (Byvaltcev et al 2023). However, recent computational modeling suggests that extracellular K^+^ accumulation alone may be insufficient to confer PAP depolarization, as rapid K^+^ clearance and membrane leak currents limit the magnitude of extracellular K^+^ elevations. These analyses instead suggest that receptor-mediated currents, including astrocytic NMDA and GABA receptor activation, may contribute more substantially to depolarization of astrocytic processes (Nakatani and De Schutter 2024).

A complementary feedback mechanism involves chloride (Cl^−^) dynamics in astrocytes. Astrocytes can function as a dynamic Cl^−^ reservoir, and GABA_A receptors localized near synaptic contacts may enable activity-dependent Cl^−^ release into the perisynaptic space (Untiet et al 2023; Untiet et al 2024). Experimental manipulation of astrocytic Cl^−^ levels indicates that this flux can influence inhibitory signaling—that is, increasing astrocytic Cl^−^ accelerates decay of neuronal Ca^2+^ signals and shortens inhibitory postsynaptic responses, whereas decreasing astrocytic Cl^−^ prolongs Ca^2+^ signals and enhances excitation during prolonged activity. These data suggest that PAPs participate in ionic feedback loops in which PAPs modulate K^+^, glutamate, and Cl^−^ homeostasis, thereby helping to shape excitatory and inhibitory balance.

Beyond these roles, PAPs can also participate in structural remodeling of synaptic circuits. The distribution and activity of glutamate transporters within PAPs are regulated by neuronal activity: increased synaptic activity can promote clustering of transporters near synapses, whereas reduced activity leads to their redistribution within astrocytic membranes (Benediktsson et al 2012). These data suggest a mechanism by which astrocytes adjust local transmitter clearance in response to changes in neuronal activity.

PAPs also exhibit localized protein synthesis. PAPs contain ribosome-bound mRNAs and perform activity-dependent translation, enabling rapid microdomain-specific remodeling of the perisynaptic proteome (Mazaré et al 2020; Sapkota et al 2022; Shishkova and Rogachevsky 2023). Blocking translation prevents activity-dependent changes in PAP-associated proteins during learning and experience-dependent plasticity (Sapkota et al 2022), suggesting that local protein synthesis contributes to structural and functional activity-dependent adaptation of astrocytic processes.

Finally, PAPs can contribute to synapse remodeling through phagocytic mechanisms. PAPs contain endosomes and phagosomes, which can account for over 60% of subcellular components within these processes (Aboufares El Alaoui et al 2021). Astrocytic engulfment involves receptors such as MEGF10 and MERKT; deletion of these receptors reduces synapse clearance by up to 85% in vivo (Chung et al 2013; Bellesi et al 2017; Lee et al 2021; Kim et al 2025). Brain region and synapse-type specificity have also been suggested, where MEGF10 eliminates hippocampal excitatory synapses in response to neuronal activity (Lee et al 2021), and in the striatum, MEGF10 and MERTK selectively remove corticostriatal synapses relative to thalamostriatal inputs (Kim et al 2025). Further, sleep deprivation enhances PAP engulfment of presynaptic terminals and elevates MERTK expression and lipid peroxidation, suggesting that metabolic stress may modulate astrocyte-dependent phagocytic activity (Bellesi et al 2017). However, the mechanisms that determine synapse selection and the conditions under which astrocyte-mediated engulfment is beneficial versus detrimental remain incompletely understood (Sokolova et al 2021).

## Beyond Synapses: PAPs as Integrative Hubs

Astrocytes function as distributed integrative units that couple local synaptic activity to broader tissue-level homeostasis (Hösli et al 2022). This integrative capacity derives from the spatial compartmentalization of their morphology: nanoscale PAPs interface with and respond to neuronal signals and shape synaptic microenvironments, while PvAPs ensheathe the vasculature to coordinate neurovascular coupling and maintain blood–brain barrier integrity (Boulay et al 2017; Mazaré et al 2020). Although molecularly and functionally distinct, PAPs and PvAPs are connected within the same astrocytic arbor, providing a structural framework through which local synaptic signals may be coupled to vascular responses (Boulay et al 2017; Mazare et al 2021).

### Coupling Synaptic Activity to the Neurovascular Unit

Emerging studies in awake animals provide evidence for this functional linkage. Spatially localized Ca^2+^ microdomains arising in fine astrocytic processes precede sensory-evoked vasodilation and neuronal excitation (Stelzner et al 2024). This astrocytic Ca^2+^ activity is thought to originate within fine astrocytic processes, including PAPs, following neurotransmitter release, raising the possibility that these domains contribute to activity sensing and feedforward signaling that anticipate metabolic demand and promote increases in cerebral blood flow. However, the mechanisms by which these Ca^2+^ microdomain signals propagate to PvAPs remain to be fully established.

In parallel, Ca^2+^-dependent ATP release through gap junctions and Panx-1 channels can modulate extracellular conversion of ATP to adenosine, which triggers secondary Ca^2+^ elevations and the release of vasoactive metabolites from PvAPs (Chen et al 2024). Although multiple studies document Ca^2+^ dynamics in fine astrocytic processes (Bindocci et al 2017; Stobart et al 2018; Arizono et al 2020; Armbruster et al 2022), the nanoscale origin, propagation rules, and communication between PAPs and PvAPs remain incompletely understood and likely require ultrafast, high-resolution imaging combined with dual-compartment recordings.

Interestingly, the finding that local translational machinery shapes the molecular repertoire of PvAPs (Boulay et al 2017; Mazaré et al 2020) raises the possibility that activity-dependent, spatially confined protein synthesis contributes to the specialization of the neuro-glial-vascular unit; however, direct links to PAP-derived signaling remain to be established. Recent TurboID-mediated, vessel-isolated proteomics provides a more comprehensive and semi-quantitative map of astrocyte endfoot proteins, offering a framework for systematic interrogation of blood–brain barrier-associated pathways (Hill et al 2025).

### PAP Interactions with the Extracellular Matrix

PAPs also integrate extracellular matrix (ECM) signals, particularly through interactions with perineuronal nets (PNNs). PNNs are dense ECM structures that primarily ensheathe the somata of primarily fast-spiking inhibitory neurons—and contain discrete structural “holes” or perineuronal gaps that permit PAP intrusion, generating microdomains where glutamate transporters, ion channels, and ECM receptors form a confined extracellular microenvironment (Tewari et al 2024). These restricted domains may act as localized compartments that help maintain low ambient glutamate levels and limit activation of extrasynaptic receptors.

Disruption of PNNs removes this spatial constraint, permitting PAP overextension, impaired K^+^ clearance, and glutamate spillover, culminating in cortical hyperexcitability (Tewari et al 2024). Thus, interactions between PAPs and the ECM represent an additional structural mechanism through which astrocytes help preserve synaptic specificity and maintain excitation–inhibition balance.

### PAP–Microglia Communication

A further layer of communication occurs between PAPs and microglia. Neuronal activity evokes extracellular ATP release from both neurons and astrocytes (Badimon et al 2020; Cserép et al 2020). Microglia detect ATP via the purinergic receptor P2RY12, directing process extension toward active neuronal soma or synapses. Once recruited, microglia rapidly catabolize ATP into adenosine, an inhibitory gliotransmitter that binds to high-affinity neuronal A1 receptors and suppresses neurotransmitter release, thereby protecting circuits from hyperexcitability (Badimon et al 2020). Given their nanometric proximity to synaptic sites, PAPs are well positioned to contribute to local ATP signaling that guides P2RY12-dependent microglial recruitment. However, direct evidence for PAP-specific ATP release remains limited, and whether this spatial arrangement meaningfully influences the local signaling landscape that guides microglial chemotaxis is unknown.

Astrocytes and microglia also cooperate in synaptic and debris clearance through partially overlapping yet distinct mechanisms. Physiological stressors such as acute sleep deprivation elicit rapid, MERTK-dependent astrocytic phagocytosis of presynaptic elements, whereas chronic sleep restriction triggers complement C3 deposition and CR3-mediated microglial engulfment (Bellesi et al 2017). Notably, microglial engagement occurs in the absence of canonical neuroinflammatory signatures, suggesting a noninflammatory primed state coordinating astrocyte–microglia interactions.

These observations raise an intriguing question of whether astrocytes and microglia participate in complementary phases of synaptic remodeling, with astrocytic processes contributing to rapid, activity-dependent clearance during transient perturbations and microglia mediating more extensive engulfment during sustained perturbations. A similar functional specialization appears during apoptotic cell clearance, where astrocytic processes engulf diffuse apoptotic fragments while microglia clear larger cellular structures (Damisah et al 2020). Although PAPs were not directly resolved in that study, the polarized remodeling described is consistent with possible involvement of fine astrocytic processes.

Microglia can also instruct PAP remodeling through signaling pathways. In sensory-deprived postnatal mouse barrel cortex, neuronal CX3CL1–microglial CX3CR1 signaling activates microglial secretion of Wnt ligands, which engage canonical Wnt signaling in astrocytes and trigger PAP retraction (Faust et al 2025). Blocking microglial Wnt release or inhibiting canonical Wnt signaling prevents PAP retraction, microglial engulfment, and synapse loss.

In other contexts, microglia can directly remodel astrocytic processes. In rats receiving a high-salt diet, reactive microglia accumulate around vasopressin neurons and phagocytose astrocytic processes, leading to reduced PAP coverage (Gu et al 2025). These distinct modes of PAP remodeling, either through instructive signaling or direct removal, likely represent separable mechanisms governing synaptic reorganization.

Finally, astrocyte-derived cytokines represent another pathway through which astrocytes influence microglial synapse remodeling. Interleukin-33 (IL-33) released from astrocytes engages IL1RL1 receptors on microglia to promote synaptic engulfment (Vainchtein et al 2018). Loss of astrocytic IL-33 reduces microglial engulfment and alters synaptic activity in several circuits. However, the subcellular origin of IL-33 release within astrocytes remains unclear, and evidence linking this pathway specifically to PAP-localized signaling is currently lacking. PAPs may contribute to the local signaling environment in which such interactions occur, but this remains to be determined.

Collectively, these studies suggest that PAPs function as integrative astrocytic microdomains linking local synaptic activity with broader homeostatic processes, including regulation of cerebral blood flow, ECM organization, and coordinated microglial surveillance. However, the precise mechanisms underlying these roles, including Ca^2+^ signal propagation, the sources of key gliotransmitters, and the signals governing transitions between astrocyte- and microglia-mediated clearance, remain important open questions.

## PAP Dysfunction in Neurodegenerative Diseases: When Communication Breaks Down

**Figure 4.**
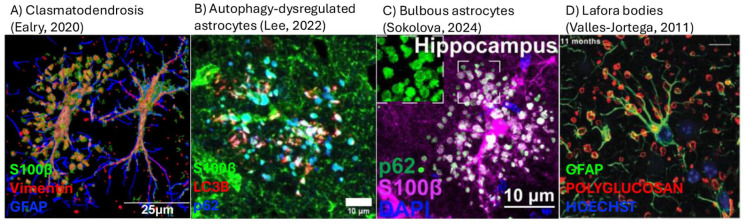
Perisynaptic astrocytic process (PAP) structural remodeling in dysfunctional contexts. (A) Clasmatodendrosis in astrocytes of the aged mouse hippocampus, characterized by beading and fragmentation of processes (with permission from Early et al 2020). (B) Autophagy-dysregulated astrocytes in the aged mouse hippocampus showing accumulation of the autophagy markers p62 and LC3B (with permission from Lee et al 2022). (C) Bulbous astrocytes in the 6-month-old hippocampus of the hAPP-NLF knock-in mouse model displaying prominent p62-positive perisynaptic accumulations (with permission from Sokolova 2024). (D) Lafora bodies (polyglucosan inclusions) present in the 11-month-old hippocampus of the malin knockout mouse model of Lafora disease (with permission from Valles-Ortega et al 2011).

Astrocytes undergo progressive structural and functional alterations across multiple neurodegenerative conditions (Figure 4). Emerging data suggest that early changes may occur at the level of fine astrocytic processes, including PAPs, which form key interfaces for neuron–glia communication at synapses. As pathological stressors accumulate, these perisynaptic compartments may become sites of convergent dysfunction involving metabolic, cytoskeletal, and immune-related pathways.

### Regionally Selective PAP Vulnerability

Across experimental models and human tissue, a recurring pathological feature is astrocytic morphological atrophy, characterized by reductions in process volume, branching complexity, and the extent of fine processes, including PAPs. These changes are observed in aging (Popov et al 2023), epilepsy (Plata et al 2018), and vulnerable brain regions in Alzheimer’s disease (AD) and Parkinson’s disease (PD) (Yeh et al 2011; Kulijewicz-Nawrot et al 2012; Ramos-Gonzalez et al 2021; Rodríguez et al 2023; Sokolova et al 2024).

The spatial distribution of these alterations suggests region-specific vulnerability. For example, PAP changes are prominent in the hippocampus during aging and following status epilepticus (Plata et al 2018; Popov et al 2023), a region characterized by high synaptic plasticity and metabolic demand (Bettio et al 2017). In AD models, PAP-associated pathology is enriched in disease-relevant regions, including the hippocampus, piriform cortex, and subiculum (Sokolova et al 2024).

In more advanced stages, astrocytes can develop clasmatodendrosis, a degenerative phenotype marked by swollen and fragmented processes, including PAPs (Figure 4A) (Balaban et al 2021). The dystrophic morphologies share features with Lafora and polyglucosan bodies, which reflect pathological glycogen accumulation associated with impaired autophagic processing (Figure 4B) (Duran 2023). Related phenotypes have been described in AD mouse models, including “bulbous” PAPs in hAPP-NL-F and hAPP-NL-G-F knock-in mice, which exhibit accumulation of p62/SQSTM1-positive autophagosomes within fine astrocytic processes (Sokolova et al 2024) (Figure 4C, D). Similar features have also been reported in “autophagy-dysregulated” astrocytes in aged, plaque-enriched stages of APP/PS1 transgenic mice (Lee et al 2022).

Notably, in the hAPP-NL-F model, these “bulbous” PAP phenotypes are enriched in regions known to be vulnerable in AD and emerge alongside early synaptic alterations, preceding detectable plaque accumulation (Sokolova et al 2024). While these observations suggest a close association between PAP alterations and synaptic pathology, whether these changes are causal drivers or early correlates of disease progression remains to be determined.

Consistent with this view, AD-associated proteomic changes are enriched in PAP-associated compartments early in disease progression and are distinct from those observed in the astrocyte soma (Griffiths et al 2025). These findings highlight the importance of compartment-resolved analyses, as commonly used astrocytic markers such as GFAP do not label fine processes and may therefore overlook early structural alterations (Ogata and Kosaka 2002).

### Metabolic Dysfunction as a Potential Driver of PAP Pathology

A growing body of data suggests that metabolic dysfunction may contribute to PAP pathology. Transcriptomic studies of human AD tissue demonstrate upregulation of genes associated with structural maintenance alongside downregulation of genes involved in mitochondrial function and endolysosomal pathways (Galea et al 2022). Proteomic analyses suggest a related trajectory, with early alterations in presynaptic metabolism and vesicle recycling, followed by changes in inhibitory synaptic proteins and later postsynaptic defects that correlate with progressive alterations in PAP-enriched astrocytic proteins (Griffiths et al 2025).

These observations raise the possibility that metabolic disturbances within PAP-associated compartments may precede and potentially contribute to synapse dysfunction; however, direct causal links remain to be established.

The accumulation of glycogen-based inclusions, including Lafora and polyglucosan bodies, further supports the presence of metabolic distress. Astrocytic glycogen serves as an important energy reservoir for neuronal activity (Suzuki et al 2011; Rothman et al 2022; Duran 2023), and its sequestration may reflect impaired metabolic support. Given that the destruction of glycogen mobilization impairs learning and memory (Suzuki et al 2011; Duran 2023), altered glycogen handling within astrocytes could contribute to synaptic vulnerability. Whether these changes are specifically localized to PAPs and how they impact synaptic function remain open questions.

### Intracellular Machinery Failure: Cytoskeletal, Ca^2+^, and Proteostatic Dysregulation

PAP alterations are also associated with disruptions in the molecular machinery required to maintain fine astrocytic structural integrity and function (Figure 3). The actin–membrane linker ezrin, enriched in PAPs, is important for process motility and structural stability. Perturbations in ezrin expression or phosphorylation state are associated with reduced process complexity, altered astrocyte–synapse apposition, and impairments in synaptic plasticity (Lavialle et al 2011; Schacke et al 2022; Chen et al 2025). In AD mouse models, reduced ezrin levels are observed in astrocytes with dysfunctional PAPs at the onset of synapse pathology (Sokolova et al 2024), while in PD models, LRRK2 G2019S-mediated ezrin phosphorylation is linked to reduced astrocytic territory and synaptic alterations (Wang et al 2023), suggesting the association of ezrin with alteration of astrocytes and PAPs in neurodegeneration. Similar principles are observed in stress paradigms, where ezrin modulation influences astrocyte morphology and behavioral outcomes (Lin et al 2023; Lin et al 2025).

Altered Ca^2+^ signaling may further contribute to PAP dysfunction. In the APP/PS1 transgenic mouse model of AD, Aβ oligomers activate astrocytic TRPA1 channels, leading to elevated Ca^2+^ activity that is associated with process retraction and neuronal dysfunction (Paumier et al 2022). These changes may influence downstream pathways, including gliotransmitter release, mGluR5 signaling, glutamate clearance, and generation of reactive oxygen species, creating a synaptic microenvironment prone to excitotoxicity (Kim et al 2025), although the relative contribution of each mechanism remains under investigation.

Disruptions in PAP-localized translation and proteostasis have also been observed. In presymptomatic APP/PS1 mice, PAP-associated transcripts linked to inflammatory and ER stress pathways are selectively upregulated and can be partially normalized by JAK-STAT3 inhibition (Avila-Gutierrez et al 2025). These findings suggest that early alterations in local protein synthesis and proteostatic regulation may contribute to disease-associated astrocyte states.

A downstream consequence of these combined perturbations is impaired glutamate clearance. Oxidative stress can promote GLT-1 mislocalization and internalization (Scimemi et al 2013), and because PAP coverage is spatially heterogeneous, this may lead to local imbalance in glutamate signaling and synaptic plasticity, as shown in 3xTg-AD mice (Brymer et al 2023). Together, these findings point to a multifactorial disruption of structural, signaling, and homeostatic mechanisms within fine astrocytic processes.

### Synapse Remodeling and Immune-Related Pathways

Alterations in PAP function are also associated with changes in astrocyte-mediated synapse remodeling. In APP/PS1 mice, inhibition of astrocytic EphA4 reduces complement C1q deposition and astrocyte-mediated synapse engulfment (Yang et al 2025). Although EphA4 localization to PAPs in adult tissue remains to be fully defined, developmental (Tremblay et al 2009) and proteomic (Soto et al 2023) data suggest enrichment at synapse-associated astrocytic compartments.

Apolipoprotein E (APOE) isoforms further modulate astrocyte-mediated synaptic engulfment. APOE2 is associated with enhanced engulfment and reduced C1q accumulation, whereas APOE4 shows the opposite pattern (Chung et al 2016). Astrocytic phagocytic responses also exhibit context-dependent plasticity. In P301S tauopathy models lacking microglial TREM2, astrocytes can compensate by engulfing inhibitory synapses (Dejanovic et al 2022), suggesting flexibility in glial contributions to synapse remodeling.

These findings indicate that dysregulation of synapse removal may arise not from the engulfment machinery itself but from altered control over when and where synapses are targeted. Synaptic “tagging” signals, such as phosphatidylserine exposure, demonstrated in both developmental (Scott-Hewitt et al 2020) and Aβ oligomer-related (Rueda-Carrasco et al 2023) paradigms, are likely contributors, although how these cues are regulated and interpreted by astrocytes remains incompletely understood.

Astrocyte–microglia coordination further shapes these processes. Human AD tissue shows increased synapse engulfment by both astrocytes and microglia (Taddei et al 2023), a phenotype also demonstrated in vitro using human cells with effects influenced by APOE genotype (Tzioras et al 2023). Although MFGE8 has been implicated in phagocytosis by astrocytes and microglia (Tzioras et al 2023), its best-established role is as a bifunctional linker between phosphatidylserine-expressing targets and phagocytes (Hanayama et al 2002; Nagata et al 2016). Notably, early in AD models, at the onset of synapse pathology, when synapses externalize phosphatidylserine (Rueda-Carrasco 2023) and bulbous PAPs with metabolic impairment emerge (Sokolova et al 2024), astrocytes increase secretion of MFG-E8, suggesting a potential role in coordinating microglia-mediated synapse engulfment. Consistent with this interpretation, astrocyte-specific deletion of MFG-E8 reduces microglia-mediated synapse elimination and synapse loss in AD mouse models (Sokolova et al 2024).

Together, these observations suggest that PAP-associated dysfunction occurs within a broader, coordinated glial network. A key unresolved question is how metabolic, synaptic, and inflammatory signals are integrated to regulate the transition from physiological synapse remodeling to pathological synapse loss and how alterations in fine astrocytic processes contribute to this shift.

## Future Directions

It is becoming increasingly clear that astrocytes, particularly their fine perisynaptic processes, including PAPs, contribute to the regulation of synaptic homeostasis. PAPs can be viewed as specialized microdomains in which biochemical signaling, local translation, metabolic flux, and structural remodeling converge to influence synaptic efficacy and circuit function. However, fundamental principles governing PAP biology remain incompletely defined. This reflects, in part, the difficulty of selectively interrogating these ultrafine structures as molecularly distinct compartments, as well as continued reliance on markers such as GFAP to infer astrocyte function, despite their inability to resolve the nanoscale, motile processes most relevant to synaptic interactions (Figure 1). Addressing these limitations will be essential for developing a mechanistic understanding of astrocyte–synapse communication in both physiological and pathological contexts.

A major priority is the development of integrated, multimodal approaches capable of resolving PAP dynamics with synapse-level precision. Progress will likely depend on combining in vivo super-resolution imaging, large-volume electron microscopy, and computational segmentation approaches to capture PAP geometry, turnover, and circuit-specific variability. In parallel, further advances are needed to profile the molecular composition of PAPs, including approaches to characterize local transcriptomes, ribosome occupancy, and nascent protein synthesis. Such datasets will be important for understanding how astrocytes generate spatially restricted responses to synaptic activity, metabolic demand, and environmental perturbations.

Further, while intact mammalian systems such as rodent models remain essential for studying cell–cell interactions, the integration of neural, glial, and vascular components, including blood–brain barrier function, and their impact on circuit functions and behavior, it will be equally important to broaden the experimental framework to include human systems. This is particularly relevant given emerging data indicating species-specific differences in PAP size, metabolic capacity, process complexity, and territorial organization (Bushong et al 2001; Oberheim et al 2009; Verkhratsky et al 2023; Hercher et al 2025). Progress toward human relevance will therefore require coordinated use of human-derived approaches alongside established rodent and other animal models, which remain critical for causal, in vivo mechanistic tests. Although organoid systems provide access to human astrocytes, many current platforms do not yet fully recapitulate mature PAP architecture and dynamics, highlighting the need for continued development of more advanced assembloid systems and rigorous ultrastructural benchmarking using super-resolution imaging and volume electron microscopy. Human glial chimeric models provide a complementary in vivo approach, enabling human astrocytes to mature within intact circuits and influence synaptic function (Mariani et al 2019; Jin et al 2026). Together, these strategies support a framework of parallel cross-species validation, rather than substitution of one model for another.

Future work will also benefit from placing PAPs within their broader multicellular context. Astrocyte interactions with microglia, the vasculature, and the ECM most likely work together to shape whether synapses are maintained, remodeled, or lost. A key challenge is to define the regulatory principles that govern these transitions. This includes understanding how changes in metabolic state, ion homeostasis, or proteostatic balance within astrocytes influence downstream signaling pathways, including those involved in synapse remodeling and glial coordination. Determining how these processes vary across circuits and disease states will be important for linking local astrocyte dysfunction to systems-level outcomes.

Ultimately, a central goal is to determine how alterations in fine astrocytic processes relate to early changes in synaptic function and vulnerability. PAPs are well positioned to reflect local circuit state and to integrate multiple forms of homeostatic demand. Clarifying how these processes respond to stress, as well as when they transition from adaptive to maladaptive states, may provide insight into early mechanisms of circuit dysfunction. Continued integration of structural, metabolic, and functional approaches across models and disease contexts will be necessary to establish how PAPs contribute to synapse stability and how their dysfunction may be linked to neurodegenerative processes.
